# Chitosan-Based Polyelectrolyte Complexes for Doxorubicin and Zoledronic Acid Combined Therapy to Overcome Multidrug Resistance

**DOI:** 10.3390/pharmaceutics10040180

**Published:** 2018-10-09

**Authors:** Simona Giarra, Silvia Zappavigna, Virginia Campani, Marianna Abate, Alessia Maria Cossu, Carlo Leonetti, Manuela Porru, Laura Mayol, Michele Caraglia, Giuseppe De Rosa

**Affiliations:** 1 Department of Pharmacy, University of Naples Federico II, D. Montesano 49, 80131 Naples, Italy; simona.giarra@unina.it (S.G.); virginia.campani@unina.it (V.C.); laumayol@unina.it (L.M.); 2 Department of Biochemistry, Biophysics and General Pathology, Second University of Naples, L. De Crecchio 7, 80138 Naples, Italy; silvia.zappavigna@unicampania.it (S.Z.); marianna.abate1991@gmail.com (M.A.); alessiacossu@libero.it (A.M.C.); michele.caraglia@unina2.it (M.C.); 3 UOSD SAFU, IRCCS Regina Elena National Cancer Institute, E. Chianesi 53, 00144 Rome, Italy; carlo.leonetti@ifo.gov.it (C.L.); manuela.porru@ifo.gov.it (M.P.)

**Keywords:** chitosan, polyelectrolyte complexes, doxorubicin, zoledronic acid, multidrug resistance

## Abstract

This study aimed to develop nanovectors co-encapsulating doxorubicin (Doxo) and zoledronic acid (Zol) for a combined therapy against Doxo-resistant tumors. Chitosan (CHI)-based polyelectrolyte complexes (PECs) prepared by ionotropic gelation technique were proposed. The influence of some experimental parameters was evaluated in order to optimize the PECs in terms of size and polydispersity index (PI). PEC stability was studied by monitoring size and zeta potential over time. In vitro studies were carried out on wild-type and Doxo-resistant cell lines, to assess both the synergism between Doxo and Zol, as well as the restoring of Doxo sensitivity. Polymer concentration, incubation time, and use of a surfactant were found to be crucial to achieving small size and monodisperse PECs. Doxo and Zol, only when encapsulated in PECs, showed a synergistic antiproliferative effect in all the tested cell lines. Importantly, the incubation of Doxo-resistant cell lines with Doxo/Zol co-encapsulating PECs resulted in the restoration of Doxo sensitivity.

## 1. Introduction

One of the main limitations of conventional chemotherapy is the development of a malignant cell’s resistance to one or more anticancer drugs. This process is known as “multidrug resistance” (MDR) which inevitably leads to a reduction of therapy effectiveness [1,2,3,4]. Generally, hydrophobic and amphipathic natural molecules, such as anthracyclines (e.g., doxorubicin (Doxo)) are more prone to developing resistance compared to other substances [5,6]. It is well known that the over-expression of some proteins of the efflux pumps ATP-binding cassette (ABC) family is one of the major causes of the MDR phenomenon [7,8,9]. One of the main components of the ABC family is represented by P-glycoprotein (P-gp), also known as MDR protein 1 (MDR1). P-gp is normally expressed in different normal tissues, such as the kidney, liver, pancreas, colon, and bone, where it is involved in the extrusion of neutral or weakly basic amphiphilic substances penetrated into the cells [10,11]. Therefore, tumors derived from these tissues have a greater expression of P-gp compared to others [12]. The function, as well as the ATPase activity of the P-gp, seems to be affected by intracellular cholesterol levels because very high levels of cholesterol have been found in the plasma membranes of MDR^+^ tumor cells [13,14,15,16]. The most frequent bone tumor observed clinically is osteosarcoma. The standard treatment for conventional osteosarcoma is based on pre- and post-operative chemotherapy, including Doxo, cisplatin, and methotrexate. Despite numerous attempts to find new therapeutic approaches for osteosarcoma, the patients’ prognosis has not improved in the last decades. Because Doxo is a substrate of P-gp, its cytotoxicity is highly limited. Both natural and synthetic inhibitors of P-gp have been tested to reverse Doxo resistance in osteosarcoma cell lines in vitro. The specific silencing of P-gp or the inhibition of pathways involved in MDR—such as the hypoxia inducible factor-1—appear to be promising strategies. Bisphosphonates, such as zoledronic acid (Zol), have been shown to reduce osteolysis induced by bone metastasis and exhibit highly selective localization and retention in bone, thus making them attractive agents in the treatment of bone metastasis. Studies have shown that Zol exerts pleiotropic anti-tumor effects against osteosarcoma cells in vitro, including antiproliferative and immunomodulatory effects. In previous studies, the authors demonstrated that Zol is a multi-target chemo-immuno-sensitizing agent, acting on both tumor cell and tumor microenvironment. In particular, nanomedicine loaded with Zol reversed the MDR phenotype by inhibiting the mevalonate pathway and the HIF-1α-dependent signaling, two events that impair the energy metabolism and the activity of ABC transporters [17,18,19]. Free Zol showed a limited in vivo antitumor effect, probably associated with its rapid clearance from the circulation with a preferential bone accumulation. These observations represent the rationale for the use of Zol, in combination with the cytotoxic drug Doxo, as the first not toxic metabolic modifier effective against MDR tumors, such as osteosarcoma. Loading of Zol into conventional liposomes resulted in low drug encapsulation efficiency (EE) (around 5%), presumably due to its hydrophilic nature associated with poor water solubility [20]. On the contrary, high Zol loading into nanovectors can be achieved by exploiting the interaction of its negative charges with a positive counterpart, for example, by using hybrid self-assembling nanoparticles [21]. However, the latter should be not suitable to also guarantee a high Doxo loading.

In recent years, polyelectrolyte complexes (PECs) have attracted a great deal of attention thanks to their low manufacturing costs, together with an easy scale-up and the absence of organic solvents [22,23]. In particular, the ionotropic gelation process leads to the spontaneous formation of PECs, as a result of the electrostatic interactions between oppositely charged components. One of the main polymers suitable for gelation process is represented by chitosan (CHI), a natural cationic polysaccharide composed of d-glucosamine and *N*-acetyl-d-glucosamine units, with well-known biodegradability, biocompatibility, and bioadhesiveness properties [24]. In an acidic environment, CHI positive charges make it suitable for electrostatic interactions with an anionic counterpart, such as sodium tripolyphosphate (TPP) [25,26,27,28,29].

In this context, the authors hypothesized that CHI could be used to prepare nanomedicine-based formulations for combined delivery of both highly loaded Zol and Doxo to overcome multidrug resistance in Doxo-resistant tumors. Thus, PECs co-loaded with Doxo and Zol were developed by means of ionotropic gelation process. Specifically, the authors investigated experimental parameters crucial to achieve PECs with a low mean diameter, narrow size distribution, stability during storage, and high Doxo/Zol encapsulation efficiency. Finally, in vitro studies to assess the possibility of a combined therapy to overcome resistance in Doxo-resistant tumor cells were carried out on wild-type and MDR variants of the two human osteosarcoma cell lines, which were selected by continuous exposure to Doxo [30]. Resistant variants showed an overexpression of P-gp (referred to as p170). The level of expression of this protein in the different cell lines was directly related to the degree of resistance.

## 2. Materials and Methods 

### 2.1. Materials

CHI with a Mn and Mw equal to 1.07 ± 0.09 × 106 Da and 1.43 ± 0.11 × 105 Da, respectively, inherent viscosity >400 mPa·s, and a degree of deacetylation ranges from 82 to 88%, and TPP were obtained from Sigma-Aldrich (St.Louis, Missouri, MO, USA). CHI molecular weight was measured by gel permeation chromatography (GPC) [24]. Poloxamer F127, an amphiphilic triblock polymer made up of hydrophilic polyethylene oxide (PEO) and hydrophobic polypropylene oxide (PPO) units (number of PEO units = 100, number of PPO units = 65), was purchased from Lutrol (Basf, Ludwigshafen, Germany). Doxo hydrochloride from 3V Chimica (Rome, Italy) and Zol monohydrate (1-Hydroxy-2-imidazol-1-ylethylidene) from U.S. Pharmacopeia Convention (Twinbrook Parkway, Rockville, Maryland, MD, USA) were used.

### 2.2. Preparations of Polyelectrolyte Complexes (PECs) 

Unloaded CHI-based PECs (named PEC) were prepared by ionotropic gelation method. Briefly, CHI was added to 10 mL of aqueous acetic acid solution (2% *v*/*v*); after complete solubilization, the pH of the resulting solution was adjusted to 4.7 with NaOH (1N) and filtration trough 0.2 µm syringe filter. The TPP solution was obtained by solubilizing TPP in 5 mL of distilled water followed by filtration trough 0.2 µm syringe filter. Afterwards, PECs were obtained by adding the anionic solution into the CHI solution and leaving them under magnetic stirring (700 rpm, at room temperature) for 30 min, to allow the cross-linking reaction. The resulted PEC suspension was purified by centrifugation at 10,000 rpm for 20 min (Hettich Zentrifugen, Tuttlingen, Germany) and kept overnight at 4 °C. Various CHI and TPP concentrations, times of interaction between them, as well as surfactant addiction to the formulation were investigated. Drug-loaded PECs were obtained by simply adding Doxo (0.4 mg/mL) to the CHI solution and Zol (0.8 mg/mL) to the TPP solution, prior to proceeding with the PEC preparation, thus leading to Zol-loaded PECs (PEC-Zol), Doxo-loaded PECs (PEC-Doxo), and Zol and Doxo co-loaded PECs (PEC-Doxo-Zol).

### 2.3. Size, Polydispersity Index (PI) and ζ Potential

The average diameters, polydispersity index (PI) and ζ potential of the obtained formulations were measured via dynamic light scattering (N5, Beckman Coulter, Brea, California, CA, USA and Nano-Z, Malvern Instruments, Malvern, UK). For the analysis, each PEC formulation was properly diluted with ultrapure water and measured at room temperature. Results were calculated as the average of five runs of three independent samples. To evaluate the PEC dimensional stability, size and potential measurements were monitored for at least 30 days, in water at 4 °C (i.e., storage conditions).

### 2.4. Doxo and Zol Encapsulation Efficiency and Yield of the PECs

The preparation yield of the PECs was calculated from previously freeze-dried formulations (0.01 atm, 24 h; Modulyo, Edwards, Waltham, Massachusetts, UK). In particular, it was gravimetrically obtained from the entire mass of recovered freeze-dried PECs. For the encapsulation efficiency (EE), the supernatant obtained after purification of the loaded PECs containing free drug(s) was submitted to quantitative analyses. In particular, the percentage of Doxo entrapped into PECs was evaluated by spectrophotometric assay (UV-1800, Shimadzu Laboratory World, Kyoto, Japan) at λ = 480 nm. The linearity of the response was verified over the concentration range 62.5–0.06 µg/mL (r^2^ > 0.99). On the other hand, Zol quantification was performed by ultra-high-performance liquid chromatography (UHPLC, Shimadzu Nexera Liquid Chromatograph LC-30AD, Kyoto, Japan), with a Gemini C18, 110 Å column (250 mm × 4.6 mm, 5 µm) at λ = 220 nm, using a mobile phase composed of 20:80 (*v*/*v*) acetonitrile:tributyl-ammonium-phosphate buffer (pH = 7). The flow rate was 1 mL/min and the run time was set at 15 min. The drug EE was calculated using the following Equation (1):

EE = (Total amount of drugs in formulations-free drugs)/(Total amount of drugs in formulations) × 100
(1)

The values of the EE (%) were collected from three different batches.

### 2.5. Cell Culture

The cancer cell lines used were wild-type human osteosarcoma cells (SAOS), wild-type human bone osteosarcoma epithelial cells (U-2 OS) and their Doxo-resistant variant (SAOS DX and U-2 OS DX, respectively). All cell lines were obtained from American Type Culture Collection (ATCC; Rockville, MD, USA) and were grown in Dulbecco’s Modified Eagle’s Medium (DMEM). Cell media was supplemented with 10% heat-inactivated fetal bovine serum, 20 mM HEPES, 100 U/mL penicillin, 100 mg/mL streptomycin, 1% L-glutamine, and 1% sodium pyruvate. Cells were cultured at a constant temperature of 37 °C in a humidified atmosphere of 5% carbon dioxide (CO_2_).

### 2.6. Cell Proliferation Assay

After trypsinization, all the cell lines were plated in 100 μL of medium in 96-well plates at a density of 2 × 10^3^ cells/well. One day later, cells were treated with free Doxo, free Zol, PEC, PEC-Doxo, PEC-Zol, and PEC-Doxo-Zol at concentrations ranging from 20 µM to 0.156 µM for Doxo and from 200 µM to 0.312 µM for Zol. Cell proliferation was evaluated by MTT assay. Briefly, cells were seeded in serum-containing media in 96-well plates at a density of 2 × 10^3^ cells/well. After 24 h of incubation at 37 °C, the medium was removed and replaced with fresh medium containing all developed formulations at different concentrations. Cells were incubated under these conditions for 72 h. Then, cell viability was assessed by MTT assay. The MTT solution (5 mg/mL in phosphate-buffered saline) was added (20 μL/well), and the plates were incubated for a further four hours at 37 °C. The MTT-formazan crystals were dissolved in 1N isopropanol/hydrochloric acid 10% solution. The absorbance values of the solution in each well were measured at 570 nm using a Bio-Rad 550 microplate reader (Bio-Rad Laboratories, Milan, Italy). The percentage of cell viability was calculated as described in Equation (2):

Cell viability = (abs sample − abs blank control)/(abs negative control − abs blank control) × 100
(2)

where abs sample is the absorbance of the treated wells, abs blank control is the absorbance of only medium without cells and abs negative control is the absorbance of the untreated cells. Then, the concentrations inhibiting 50% of cell growth (IC_50_) were obtained and these values were used for subsequent experiments. MTT assay was carried out by triplicate determination on at least three separate experiments. All data are expressed as mean ± SD. 

### 2.7. Evaluation of Synergism

The evaluation of synergism was performed using dedicated software CalcuSyn, version 1.2.1 (Biosoft, Ferguson, MO, USA), which measured the interaction between the drugs by calculating the indexes of combination (CIs). CI values <1, 1, and >1 indicate synergism, additive, and antagonism, respectively. Drug combination studies were based on concentration–effect curves generated as a plot of the fraction of unaffected (surviving) cells versus drug concentration after 72 h of treatment. Assessment of synergy was performed quantifying the drug interaction by the CalcuSyn computer program (Biosoft, Ferguson, MO, USA).

## 3. Results and Discussion

### 3.1. PECs Preparation and Characterization 

CHI-based PECs were obtained through ionotropic gelation technique thanks to the ability of the amine functional groups of CHI to be protonated in acidic environment, thus providing -NH3^+^ groups able to interact with negatively charged groups of TPP [31]. Generally, oppositely charged macromolecules aggregate due to their high charge density fluctuation in solutions [22]. Therefore, PEC formation and stability are affected by several factors [32]. Among these, CHI and TPP concentrations used during the preparation process play an important role. The different polymer concentrations used, the size, and PI of the prepared PEC formulations are shown in Table 1. In all the formulations, the volume ratios between CHI and TPP phases were fixed at 2:1.

Results showed that PECs with the smallest size (in the range from ~130 to ~180 nm) were obtained using the same concentrations of both CHI and TPP. By increasing the TPP/CHI ratio, the PEC size significantly increased. This could be probably ascribed to the excess of TPP in the solution; negative charges of TPP might interact with free amino groups of pre-formed PECs, leading to PEC aggregation. A similar size increase was observed by increasing CHI concentration. This effect was probably due to a higher viscosity of the resulting solution, which slowed down the cross-linking reaction between the polymer chains, with the consequent formation of aggregates. Concentrations greater than 0.5 mg/mL of both components led to the formation of visible macro-aggregates (data not shown). The time of interaction between CHI and the cross-linking agent was found to influence PEC size and PI. More specifically, the optimal CHI and TPP concentrations, which led to the smallest PEC size, were used to prepare PECs with controlled-precipitation flow rate (Q). The results of dimensions, PI, and ζ potential analyses of PEC formulations, prepared by using CHI and TPP concentrations and different Q, are summarized in Table 2. In all formulations, the inner diameter of the syringe used for the precipitation of TPP onto the CHI solution was set at 11.99 mm.

As it can be observed, PECs obtained using a lower flow rate showed a smaller diameter. This size trend could be attributed to the time needed for the cross-linking reaction; thus, at lower flow rate, the possibility to achieve a homogeneous distribution of the polymer chains should be greater. This should promote their electrostatic interactions and the formation of PECs with a very small diameter (around ~100 nm). A positive charge, evaluated by ζ potential analysis, was found in all the formulations due to the presence of CHI primary free amino groups. On the basis of these results, the formulation named PEC(CHI 0.3-TPP 0.3)-B, obtained by using a concentration of 0.3 mg/mL of both CHI and TPP and a flow rate of 133.3 µL/min, presented optimal features in terms of mean diameter, although with a high PI (~0.4) indicating a poor homogeneity of the PEC dispersion (see Table 2). For this reason, the authors added the multi-block surfactant copolymer Poloxamer F127, at different concentrations, to the CHI acetic acid solution prior to PEC formation [33]. Table 3 shows the F127 concentrations used to prepare different PEC formulations; in all cases, the concentrations of both CHI and TPP used were 0.3 mg/mL. As expected, the addition of F127 resulted in significant PI reduction, depending on the Poloxamer concentration. In particular, in the case of the PEC(CHI 0.3-TPP 0.3)-B formulation, the addition of F127 at 10% (*w*/*w*) allowed more monodisperse PECs (PI < 0.25) to be produced, without significant change in size and ζ potential values (see Table 3).

### 3.2. Preparation and Characterization of PECs Encapsulating Doxo and Zol 

Doxo and Zol were loaded into PEC formulations, to obtain a co-delivery of these drugs for a combined therapy. Doxo and Zol EE and preparation yield of different formulations are summarized in Table 4; in all cases, CHI (0.3 mg/mL) and TPP (0.3 mg/mL) were used.

As shown in Table 4, the prepared formulations were characterized by a high yield, greater than 75% in all cases. Zol and Doxo EE were found to be similar in PECs loaded with one or both drugs. In particular, more than 20% of the initial loaded Doxo was found in the PECs. Surprisingly, Zol showed a very high EE (>80%) into formulation, also in association with Doxo. This result was probably due to its electrostatic interaction with positive charges of CHI, thus resulting in a more stable encapsulation. On the other hand, Doxo encapsulation should be related to its interaction with TPP. The mean size as well as the PI of PECs loaded with one or both drugs, were slightly increased (see Table 5).

### 3.3. Stability Studies

**Figure 1:**
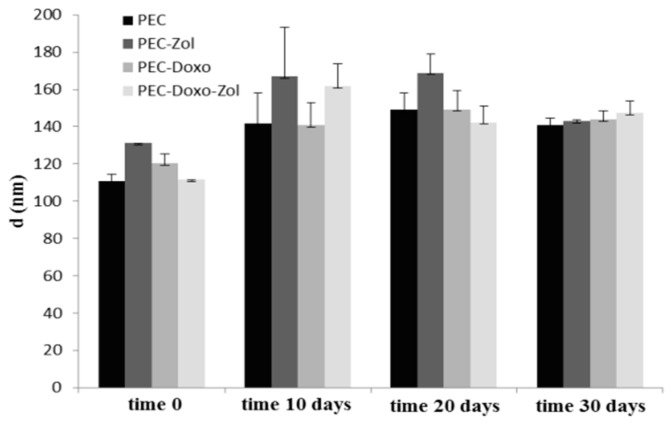
Size and PI of different PEC formulations as a function of time, in water at 4 °C.

In order to evaluate the stability of prepared formulations in storage conditions, the mean diameters, PI, and ζ potential were analyzed as a function of time, in water at 4 °C. As shown in Figure 1, all formulations underwent a slightly increase in size after 10 days; they then had a narrow size distribution for up to 30 days. Moreover, for all analyzed formulations, ζ potential values remained stable for up to 30 days (data not shown).

### 3.4. Cell Proliferation Assay 

**Figure 2:**
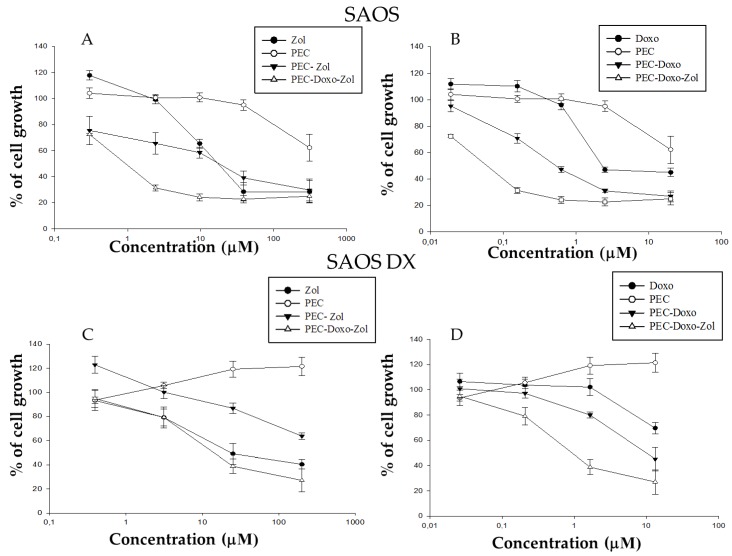

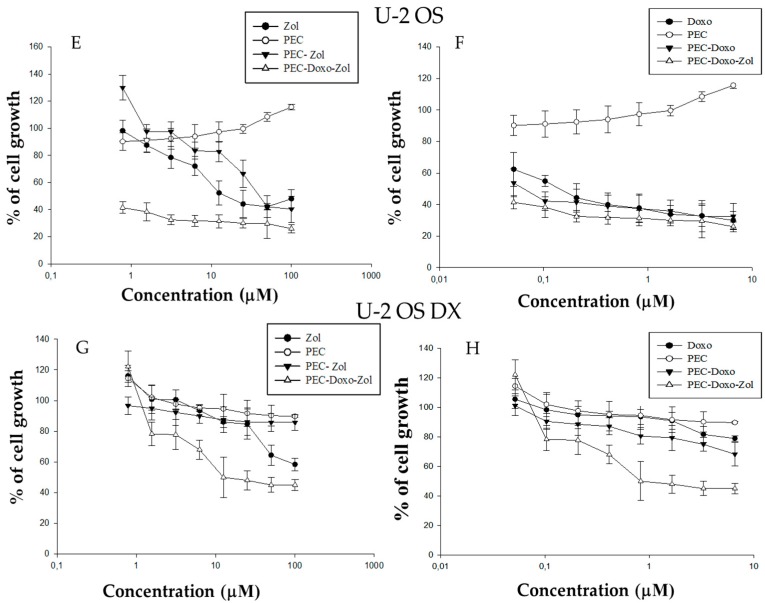
Dose–effect relationship of all developed formulations on wild-type and Doxo-resistant wild-type human osteosarcoma cells (SAOS) and wild-type human bone osteosarcoma epithelial cells (U-2 OS) proliferation. Results are expressed as % of cell growth vs. concentration (μM) of Zol (**A**, **C**, **E**, and **G**) or Doxo (**B**, **D**, **F**, and **H**). In the case of cells treated with unloaded PECs, the PEC concentration was adjusted as equivalent to the concentration of drug-loaded PECs.

The effects of free Doxo, free Zol, PEC, PEC-Doxo, PEC-Zol, and PEC-Doxo-Zol were evaluated on the proliferation of wild-type SAOS, wild-type U-2 OS, SAOS DX, and U-2 OS DX cancer cell lines by MTT assay. All the tested formulations induced a dose-dependent growth inhibition in all the cell lines analyzed after 72 h, whereas treatment with unloaded PECs did not produce significant cytotoxic effects (see Figure 2).

The results of IC_50_ after 72 h of treatment are shown in Table 6.

The IC_50_ values of free Doxo were equal to 2 μM and superior to 20 μM for wild-type and SAOS DX cells, whereas they were equal to 0.14 μM and superior to 6.60 μM for wild-type and U-2 OS DX cells (see Table 6). The PEC-Doxo induced a 50% growth inhibition at a concentration of 0.5 μM and 10.4 μM for wild-type and SAOS DX, respectively, whereas the concentration was 0.06 μM and superior to 6.60 μM for wild-type and U-2 OS DX, respectively (see Table 6). These data demonstrated that the PECs, even without the co-encapsulation of Zol, were able to strongly increase the cytotoxicity of Doxo in all the tested formulations, except in U-2 OS DX cells. It is noteworthy that Doxo encapsulation in other nanocarriers (e.g., stealth liposomes) results in a reduced Doxo cytotoxicity [34,35,36]. As previously reported by other authors for different Doxo-encapsulating nanovectors, the cell uptake of Doxo encapsulated into PECs should occur by endocytosis, whereas free Doxo enters cancer cells primarily through passive diffusion across the plasma membrane [37]. Taking into account that unloaded PECs are not cytotoxic at the experimental conditions used here, the enhanced cell toxicity observed with PEC-Doxo should be reasonably due to the enhanced Doxo intracellular concentration. Previously, other authors have demonstrated the possibility of increasing cell apoptosis by modulating Doxo intracellular trafficking [37]. On the other hand, the PEGylated nanocarriers have been shown to reduce drug uptake into the target cells [38]. The IC_50_ values of free Zol were equal to 17 μM for wild-type SAOS and 23.4 μM for SAOS DX, whereas they were equal to 15.60 μM for wild-type U-2 OS and superior to 100 μM for U-2 OS DX cells. The encapsulation of Zol in PECs did not potentiate its antitumor activity; in fact, the IC_50_ values for PEC-Zol were 16 μM and >200 μM for wild-type and SAOS DX, respectively, whereas they were 40.40 μM and superior to 100 μM for wild-type and U-2 OS DX, respectively. These results are in contrast with the authors’ previous finding in which different lipid-based nanocarriers encapsulating Zol were useful to increase Zol uptake in different cancer cell lines [20,21]. When incubating wild-type SAOS and U-2 OS with PEC-Zol in this study, a similar or enhanced cytotoxicity was found, when compared to free Zol. This could be ascribed to the strong interaction between CHI and Zol that slows down the delivery of the bisphosphonate into the cytoplasm. Further studies are needed to understand the disappearance of Zol toxicity when incubating cells with Zol-PEC. However, it is noteworthy that PEC-Doxo-Zol inhibited 50% of cell growth at a concentration of 0.05 μM and 0.9 μM for Doxo and 0.8 μM and 13.8 μM for Zol for wild-type and SAOS DX, respectively. On the other hand, it inhibited 50% of cell growth at a concentration inferior to 0.05 μM and 0.80 μM for Doxo and inferior to 0.78 μM and equal to 13.60 μM for wild-type and U-2 OS DX, respectively, by significantly enhancing the effects of both the drugs. 

### 3.5. Evaluation of Synergism 

The synergism between Doxo and Zol in PECs was calculated by using the dedicated software CalcuSyn. CI values are shown in Table 7.

As summarized in Table 7, a synergic effect was found in wild-type U-2 OS cells, whereas a strong synergism was found in wild-type SAOS, SAOS DX, and U-2 OS DX cells when co-encapsulating Doxo with Zol. Interestingly, the data on SAOX DX and U-2 OS DX strongly confirm the authors’ previous finding on the association of Doxo and Zol to revert resistance to Doxo. In particular, Zol is a multi-target chemo-immuno-sensitizing agent, acting on both tumor cell and tumor microenvironment. In fact, nanoparticles (NPs) encapsulating Zol reversed the resistance towards P-gp substrates by decreasing the synthesis of cholesterol, which is critical for the activity of P-gp and the activity of Ras/ERK1/2/HIF-1α-axis, which mediates the transcription of P-gp [19]. In this study, a synergic effect was also found in wild-type cells, thus suggesting that other mechanisms, different from the inhibition of the P-gp, could be produced when associating these two drugs.

## 4. Conclusions

In this study, CHI-based PECs co-loaded with Doxo and Zol were successfully prepared with a simple and easily up-scalable method. The results showed that polymer concentration, times of interaction between polymer/cross-linking agent, and surfactant addiction to the formulation are crucial for PEC formation and for their technological features. Finally, this study demonstrated two crucial advantages of PEC-encapsulating Doxo. First, PECs significantly enhance Doxo cytotoxicity, probably due to an enhanced internalization into the cells. Second, the authors’ hypothesis was confirmed because PECs co-administrating Zol and Doxo resulted in a significant restoration of cell sensitivity to Doxo, thus providing a promising approach to overcoming MDR. 

## Figures and Tables

**Table 1:** Size and polydispersity index (PI) of different polyelectrolyte complex (PEC) formulations. All results are expressed as mean ± SD of at least three independent experiments. CHI: chitosan TPP: tripolyphosphate.

Formulation	(CHI) mg/mL	(TPP) mg/mL	D (nm)	PI
PEC(CHI 0.4-TPP 0.5)	0.4	0.5	236.9 ± 31.2	1.14 ± 0.5
PEC(CHI 0.3-TPP 0.5)	0.3	0.5	231.9 ± 9.50	0.23 ± 0.1
PEC(CHI 0.5-TPP 0.4)	0.5	0.4	272.1 ± 86.7	0.42 ± 0.1
PEC(CHI 0.5-TPP 0.3)	0.5	0.3	314.2 ± 14.5	1.03 ± 0.6
PEC(CHI 0.5-TPP 0.5)	0.5	0.5	132.7 ± 11.2	0.43 ± 0.1
PEC(CHI 0.4-TPP 0.4)	0.4	0.4	185.1 ± 0.81	0.27 ± 0.1
PEC(CHI 0.3-TPP 0.3)	0.3	0.3	132.3 ± 6.80	0.42 ± 0.1
PEC(CHI 0.3-TPP 0.5)	0.3	0.4	287.8 ± 15.4	0.15 ± 0.1

**Table 2:** Size, PI, and ζ potential values of different PEC formulations. All results are expressed as mean ± SD of at least three independent experiments.

Formulation	(CHI) mg/mL	(TPP) mg/mL	Q (µL/min)	d (nm)	PI	ζ Potential (mV)
PEC(CHI 0.5-TPP 0.5)-A	0.5	0.5	500	223.3 ± 3.8	0.67 ± 0.3	18.1 ± 1.5
PEC(CHI 0.5-TPP 0.5)-B	0.5	0.5	133.3	187.0 ± 9.1	0.45 ± 0.2	21.3 ± 1.1
PEC(CHI 0.4-TPP 0.4)-A	0.4	0.4	500	147.7 ± 2.9	0.49 ± 0.1	19.1 ± 1.9
PEC(CHI 0.4-TPP 0.4)-B	0.4	0.4	133.3	129.4 ± 2.7	0.42 ± 2.7	19.1 ± 0.8
PEC(CHI 0.3-TPP 0.3)-A	0.3	0.3	500	127.5 ± 2.2	0.51 ± 0.3	17.8 ± 2.9
PEC(CHI 0.3-TPP 0.3)-B	0.3	0.3	133.3	104.4 ± 1.4	0.41 ± 0.3	21.4 ± 1.9

**Table 3:** Size, PI, and ζ potential values of different PECs. All results are expressed as mean ± SD of at least three independent experiments.

Formulation	(CHI) mg/mL	(TPP) mg/mL	F127 (% *w*/*w*)	d (nm)	PI	ζ Potential (mV)
PEC(CHI 0.3-TPP 0.3)-B20	0.3	0.3	20	114.1 ± 5.1	0.48 ± 0.1	17.2 ± 2.5
PEC(CHI 0.3-TPP 0.3)-B16	0.3	0.3	16	114.8 ± 6.9	0.32 ± 0.2	16.9 ± 2.1
PEC(CHI 0.3-TPP 0.3)-B10	0.3	0.3	10	110.8 ± 3.5	0.23 ± 0.2	21.2 ± 3.3

**Table 4:** Doxorubicin (Doxo) and zoledronic acid (Zol) encapsulation efficiency (EE) (%) and yield (%) of different formulations prepared. All results are expressed as mean ± SD of at least three independent experiments.

Formulation	(Zol)-Loaded (mg/mL)	(Doxo)-Loaded (mg/mL)	EE Zol (%)	EE Doxo (%)	Yield (%)
PEC	-	-	-	-	80.8 ± 0.1
PEC-Zol	0.8	-	92.3 ± 5.3	-	77.8 ± 1.3
PEC-Doxo	-	0.4	-	25.8 ± 0.5	79.4 ± 0.1
PEC-Doxo-Zol	0.8	0.4	83.1 ± 11	29.2 ± 6.6	81.6 ± 0.1

**Table 5:** Size, PI, and ζ potential values of different loaded PEC formulations.

Formulation	d (nm)	PI	ζ Potential (mV)
PEC-Zol	131.1 ± 0.1	0.32 ± 0.1	20.2 ± 1.3
PEC-Doxo	120.5 ± 4.7	0.42 ± 0.4	22.9 ± 1.6
PEC-Doxo-Zol	111.5 ± 0.1	0.39 ± 0.4	23.1 ± 2.3

**Table 6:** IC_50_ (M) of all developed formulations on wild-type and Doxo-resistant SAOS and U-2 OS, after 72 h of treatment.

**SAOS**	**IC_50Zol_**	**SAOS**	**IC_50Doxo_**
Zol	17	Doxo	2
PEC-Zol	16	PEC-Doxo	0.5
PEC-Doxo-Zol	0.8	PEC-Doxo-Zol	0.05
PEC	-	PEC	-
**SAOS DX**	**IC_50Zol_**	**SAOS DX**	**IC_50Doxo_**
Zol	23.4	Doxo	>20
PEC-Zol	>200	PEC-Doxo	10.4
PEC-Doxo-Zol	13.8	PEC-Doxo-Zol	0.9
PEC	-	PEC	-
**U-2 OS**	**IC_50Zol_**	**U-2 OS**	**IC_50Doxo_**
Zol	15.60	Doxo	0.14
PEC-Zol	40.40	PEC-Doxo	0.06
PEC-Doxo-Zol	<0.78	PEC-Doxo-Zol	<0.05
PEC	-	PEC	-
**U-2 OS DX**	**IC_50Zol_**	**U-2 OS DX**	**IC_50Doxo_**
Zol	>100	Doxo	>6.60
PEC-Zol	>100	PEC-Doxo	>6.60
PEC-Doxo-Zol	13.60	PEC-Doxo-Zol	0.80
PEC	-	PEC	-

**Table 7:** CIs values between Doxo and Zol on wild-type and Doxo-resistant SAOS and U-2 OS cells.

Cell Lines	CI_50_	Interpretation
SAOS	0.3	Strong Synergism
SAOS DX	0.4	Strong Synergism
U-2 OS	0.7	Synergism
U-2 OS DX	0.3	Strong Synergism

## Abbreviations

PECs	polyelectrolyte complexes
Doxo	doxorubicin
Zol	zoledronic acid
MDR	multidrug resistance
ABC	ATP-binding cassette family
P-gp	P-glycoprotein
CHI	chitosan
TPP	sodium tripolyphosphate
SAOS	wild-type human osteosarcoma cells
SAOS DX	Doxo-resistant human osteosarcoma cells
U-2 OS	wild-type human bone osteosarcoma epithelial cells
U2-OS DX	Doxo-resistant human bone osteosarcoma epithelial cells
GPC	gel permeation chromatography
PEO	polyethylene oxide
PPO	polypropylene oxide
PI	polydispersity index
EE	encapsulation efficiency
UHPLC	ultra-high-performance liquid chromatography
IC_50_	concentration inhibiting 50% of cell growth
CIs	indexes of combination
Q	precipitation flow rate
